# A qualitative analysis examining intersectional stigma among young adults living with HIV in Atlanta, Georgia

**DOI:** 10.1371/journal.pone.0289821

**Published:** 2023-08-10

**Authors:** Madeleine H. Goldstein, Shamia Moore, Munira Mohamed, Rosalind Byrd, Michael G. Curtis, Whitney S. Rice, Andres Camacho-Gonzalez, Brian C. Zanoni, Sophia A. Hussen

**Affiliations:** 1 School of Medicine, Emory University, Atlanta, GA, United States of America; 2 Children’s Healthcare of Atlanta, Atlanta, GA, United States of America; 3 Department of Global Health, Rollins School of Public Health, Emory University, Atlanta, GA, United States of America; 4 College of Arts and Sciences, Emory University, Atlanta, GA, United States of America; 5 Department of Behavioral, Social and Health Education Sciences, Rollins School of Public Health, Emory University, Atlanta, GA, United States of America; Human Sciences Research Council, SOUTH AFRICA

## Abstract

HIV-related stigma is a barrier to engagement in care for young adults living with HIV. Other intersecting forms of stigma (e.g., racism, sexism, homophobia), may worsen HIV-related stigma and impact engagement in care. From November 2020 to February 2021, we conducted 20 in-depth qualitative interviews among young adults living with HIV attending a large, comprehensive HIV care center in Atlanta, Georgia. Semi-structured interview guides based on Earnshaw and Chaudoir’s HIV Stigma Framework and the theory of intersectionality facilitated discussion around experiences with various forms of stigma and its possible influence on healthcare engagement. Using the social-ecological model, we used thematic analysis to contextualize how young adults living with HIV experienced intersectional stigma and enacted, anticipated, and internalized HIV stigma in both healthcare and non-healthcare settings. Most participants identified as male, Black/African American, and gay. Participants described stigma at intrapersonal, interpersonal, clinic, and community levels. Intrapersonal stigma was associated with delayed care seeking, isolation, and fear of disclosure. Interpersonal stigma included discrimination from family and friends and avoidance of close relationships to elude disclosure. At the clinic level, stigma included negative experiences with staff in HIV and non-HIV healthcare settings, which contributed to decreased engagement in care. Stigma in the community included differential treatment from employers, community leaders, and religious community and was associated with feelings of helplessness related to current societal inequalities. Coping/motivating mechanisms for stigma included prioritizing health, eliciting support from the medical care team and peers. Our findings show different intersecting stigmas are barriers to healthcare at multiple levels for young adults living with HIV, potentially exacerbating existing health and social disparities. To improve engagement in care among young adults living with HIV, future interventions should address the different mechanisms of stigma at community, clinic, interpersonal and intrapersonal levels by enhancing social support and improving healthcare structural competency.

## Introduction

HIV disproportionately impacts adolescents and young adults. In 2019, approximately 60% of new HIV infections in the United States (US) occurred among adolescents and young adults ages 13 to 34 years [1]. Young adults living with HIV face a multitude of social and logistical challenges, often leading to suboptimal rates of engagement in HIV care (defined as retention in care, adherence to medications, and viral suppression) [2]. When compared to any other age group, adolescents and young adults are the least likely to know their status and the least likely to be virally suppressed [1, 3].

Stigma has been identified as a key barrier to HIV care engagement and overall wellbeing among people living with HIV, including young adults living with HIV [4–7]. Stigma, defined as "an attribute, behavior, or reputation which is socially discrediting in a particular way,” is a complex phenomenon that operates through multiple levels and mechanisms to impact physical and mental health [8]. Earnshaw & Chaudoir’s HIV Stigma Framework conceptualizes how different mechanisms of stigma, including enacted, anticipated, and internalized stigma, can each impact different HIV-related care outcomes from well-being to retention in care to medication adherence [9, 10]. Enacted stigma refers to the unfair treatment or discrimination experienced by individuals living with HIV [10]. Anticipated stigma describes the belief that negative societal perceptions of HIV exist and the expectation that future prejudice, discrimination, or stereotyping may occur [10]. Internalized stigma is the acceptance of these negative societal attitudes regarding HIV as part of one’s own values and beliefs [10]. To date, there is a limited understanding of the different mechanisms of stigma experienced by young adults living with HIV specifically and how these mechanisms might differentially impact engagement in HIV care in this age group [4, 11].

Additionally, work to understand stigma among young adults living with HIV must account for differing racial/ethnic and sexual orientation backgrounds. Profound disparities exist within the US youth HIV epidemic, with most new diagnoses occurring among young Black or Hispanic gay, bisexual and other men who have sex with men [1]. When applied to people living with HIV, the intersectionality framework suggests that HIV-related stigma is exacerbated among marginalized groups due to the convergence of multiple stigmatized identities (e.g., racial minority identity, sexual minority identity, HIV-related identity) [12, 13]. Previous studies have shown that intersectional stigma is associated with social exclusion/isolation, decreased disclosure, and decreased access to health services, employment, and education among adult men and women living with HIV in the United States, Canada, and Indonesia [14–16].

HIV-related stigma is not only complicated by its intersection with other forms of stigma, but also by its interaction across multiple levels of stigma. Originally conceived as a transdisciplinary framework, the social-ecological model aims to understand the multiple levels (e.g., physical and social environments) that impact health beyond one’s individual characteristics [17, 18]. Prior research on HIV-related stigma has typically focused on intrapersonal factors and perceptions of interpersonal relationships, including Sandelowski et al. who found that women living with HIV in the United States experienced different mechanisms of HIV-related stigma and this contributed to a fear of social rejection, discrimination, and violence among their relationships with their children, partners, relatives, friends, employers, coworkers, and health care providers [19]. Few studies have considered a multilevel approach when investigating stigmatizing processes among individuals living with HIV, despite its potential to help identify the social, environmental, and individual factors that influence intersectional stigma and its impact on HIV care continuum outcomes. One study by Logie et al. found that women living with HIV in Canada experienced overlapping, multilevel forms of stigma and discrimination across micro (intra/interpersonal), meso (social/community), and macro (organizational/political) realms [14]. Similarly, a case study analysis by Embleton et al. described how adolescents living with HIV in sub-Saharan Africa experience intersectional stigma across multiple levels of the social-ecological model (e.g., intrapersonal, interpersonal, structural/organizational, and community levels) and its negative impact on the uptake and delivery of HIV prevention and treatment services [20]. Framing intersectional stigma within the context of the social-ecological model could lead to multi-level and multi-component evidence-based interventions that meaningfully reduce stigma and discrimination that impact HIV care continuum outcomes.

There is limited research examining these intersecting stigmas within the context of the social-ecological model, specifically among young adults living with HIV. To further contextualize and address the health inequities faced by young adults living with HIV, we sought to build on the current literature by qualitatively examining mechanisms of intersecting stigmas experienced by young adults. We further sought to examine whether young adults living with HIV perceived any relationship between these stigmas and their ability to engage in HIV care.

## Methods

### Parent study

This analysis is derived from a mixed‐methods prospective, observational cohort study evaluating healthcare transition among youth at a large HIV care center in Atlanta, Georgia, US [21–23]. Between August 2016 and June 2018, 70 participants were recruited from the pediatric clinic within 3 months prior to anticipated healthcare transition to adult care. Participants completed a self-administered baseline survey that included demographic information during their enrollment visit. Participants were followed over the subsequent year with serial surveys and medical chart abstractions in order to determine HIV care continuum outcomes and to examine their experiences throughout this process.

### Study design

From November 2020 to February 2021, we recruited a subsample of 20 young adults living with HIV to participate in this qualitative sub-study consisting of one in-depth interview. Inclusion criteria for participants in this study were being 18 years of age or older, English-speaking, and living with HIV. We contacted a total of 53 participants via telephone call or text message who had indicated interest in the parent study in being contacted for follow up studies. Of participants contacted, 29 were not responsive or available to interview, and 4 declined to participate. Enrollment continued until thematic saturation was met (e.g., no new themes or codes were identified). The study team selected participants based on participant availability/interest and a purposive sampling strategy, with specific efforts to include women and young adults with vertically acquired infection to ensure diversity of responses. We adhered to the COnsolidated criteria for REporting Qualitative research (COREQ) guidelines (S1 Appendix) for this work [24].

### Ethical statement

We obtained verbal informed consent from each participant prior to engaging in any study activities. Documentation of verbal consent was noted on a form and witnessed by the author obtaining consent (MG) and kept in the participant’s study record. The Emory University Institutional Review Board and Grady Research Oversight Committee approved this study.

### Analysis

One English-speaking, doctorate-level, female researcher (author MG) conducted and digitally audio-recorded the interviews, which averaged 1.5 to 2 hours in length. The interviewer had no pre-existing relationship with the participants prior to the study and emphasized confidentiality, openness, and honesty throughout the study. In-depth interviews (IDIs) were conducted and recorded over Zoom, a Health Insurance Portability and Accountability Act (HIPAA)-compliant videoconference platform. Both the interviewer and participants were located in a separate, private room either within their home or office at the time of the interview. The semi-structured interview guide was based on Earnshaw and Chaudoir’s HIV Stigma Framework as well as the theory of intersectionality [9, 10], and included open-ended questions within the following conceptual domains: (1) General Life Questions (including current living and work situations), (2) The Meaning of Stigma, (3) Enacted Stigma, (4) Internalized Stigma, (5) Anticipated Stigma, and (6) Intersectional Stigma. Our study team developed and then piloted the interview guide with two members of a youth advisory board comprised of young Black gay men living with HIV. The guide was iteratively revised based on their feedback. After the interview, participants were compensated with a $40 gift card.

Two analysts (MG and MM) transcribed the interviews verbatim and imported the transcripts into MAXQDA 2020 qualitative software (Berlin, Germany) for coding and thematic analysis [25]. A team-based coding approach was used with four analysts (MG, MM, SM, and RB) to enhance reliability and internal validity [26]. The research team used both deductive codes (e.g., internalized stigma, enacted stigma, and anticipated stigma) based on the guiding theoretical frameworks, as well as inductive codes (e.g., coping mechanisms, barriers to care, and social support) derived from the data to develop the codebook. Analysts then coded a subset of transcripts (N = 4) in parallel and compared the coded text to ensure consistency in application. The codebook was modified through an iterative process, including reading transcripts, recognizing recurring themes, refining code definitions, and discussing differences between coders with the research team until consensus was reached. Once the codebook was finalized, the study team then divided and independently coded the remaining transcripts. Next, we wrote thick descriptions of each theme, which were detailed analytic memos for each code explaining depth, breadth, context, and nuance [27]. Throughout this process, analysts met regularly to discuss emerging patterns and relationships in the data, including comparing the frequency and content of codes and themes. During analysis, we noted that the impacts of different kinds of stigma were described on different socio-ecological levels (e.g., intrapersonal, interpersonal, clinic, and community) [17]; the results below are therefore organized according to these levels.

## Results

### Demographics

We interviewed 20 young adults living with HIV between the ages of 27 and 29 (Table 1). The mean age of participants was 28.5 years (standard deviation = 0.4 years). Sixteen (80%) participants were male, and 17 (85%) acquired HIV horizontally (see Table 1 for summary of demographics). Most participants identified as Black/African American (19/95%) and gay (15/75%).

**Table 1:** Participant demographics.

Demographic	N = 20 (%)
Mean age (years)	28.5 ± 0.4
Gender: n (%)	
Cisgender male	16 (80.0)
Cisgender female	4 (20.0)
Race: n (%)	
Black / African-American	19 (95.0)
White/Caucasian	1 (5.0)
Sexual Orientation: n (%)	
Heterosexual/straight	3 (15.0)
Gay	15 (75.0)
Bisexual	2 (10.0)
Mode of Transmission: n (%)	
Vertical^a^	3 (15.0)
Horizontal^b^	17 (85.0)
Education Level: n (%)	
Less than high school	2 (10.0)
High school diploma or GED	5 (25.0)
Some tech school/college	7 (35.0)
Tech school/college graduate	6 (30.0)

^a^ Vertical transmission includes acquisition of HIV from a mother to her child during pregnancy, labor, delivery, or during breastfeeding.

^b^ Horizontal transmission includes acquisition of HIV from modes other than from a mother to her child during pregnancy, labor, delivery, or during breastfeeding, including through sexual contact, infected blood products, or needles.

### Overview of themes

**Fig 1:**
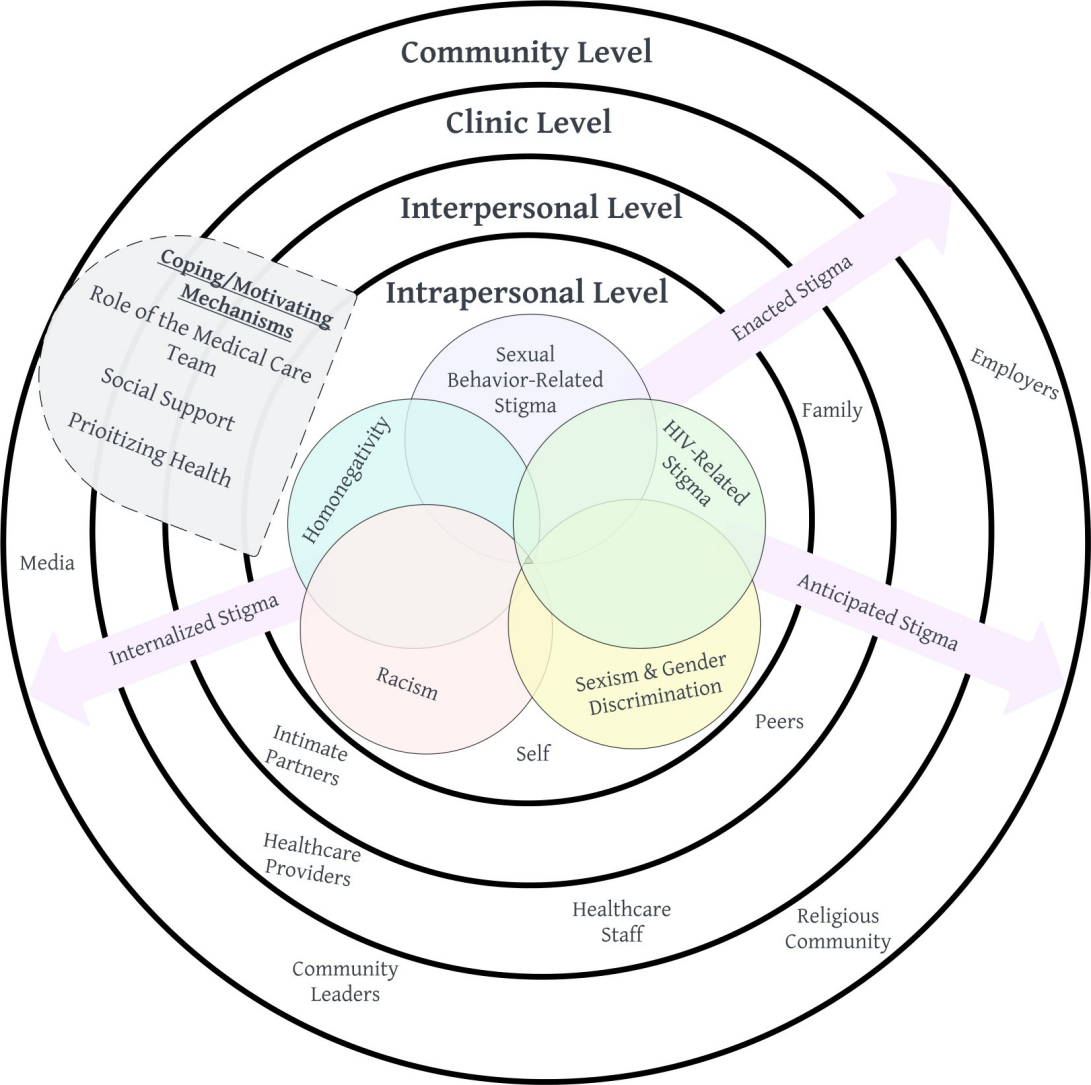
Conceptual model of intersectional stigma and coping/motivating mechanisms across different socio-ecological levels among young adults living with HIV in Atlanta, Georgia.

Participants experienced different mechanisms of stigma related to their HIV status and other aspects of their social identities (e.g., race, sexual orientation, and sexual behaviors) in both healthcare and non-healthcare settings. While these were often discussed in isolation during interviews, participants frequently reported experiences in which other forms of stigma occurred together with HIV stigma. These intersecting stigmas were often discussed at different socio-ecological levels, including intrapersonal, interpersonal, clinic, and community levels (Fig 1).

### Intrapersonal level

Internalized stigma, and to a lesser extent anticipated stigma, were the main mechanisms operating at the intrapersonal level. Many participants described their experiences with internalized stigma, as they reflected on how their feelings about HIV and their identities had changed over time, largely for the better. Many participants held negative views of HIV prior to their own diagnoses, which for some created an obstacle to engaging in HIV care; consequent feelings of shame and anticipated stigma prevented them from attending clinic appointments and/or taking their medications. As illustrated by the following quote, one participant explained his fears about attending clinic after his diagnosis:

*I felt like at that time—again*, *this is my own ignorance*, *I felt like HIV had a look*. *Again*, *that goes into that stigma*. *I felt that I could just tell or sense*. *Also*, *too*, *I was very*, *at that time*, *very ashamed*. *I knew what the clinic was*. *I knew that anybody that walked through that door*, *had what I had*, *and I didn’t want to be grouped in that category*. *I didn’t want to be grouped*. *I feared not being viewed as an individual*… (Participant 7, a 28-year-old male with horizontally acquired HIV)

For several participants, internalized HIV stigma was inextricably linked to internalized homonegativity, and contributed to shame and guilt surrounding their HIV diagnosis. Participants describe how they eventually thought that being both Black and gay, meant that they would inevitably contract HIV:

*Within our African American gay community […] one of my friends had said that*, *"Well*, *it seems like everybody in our community has it*.*" And you know*, *at first*, *I kind of laughed it off*, *but I had to think about it*. *It hurt a little bit*, *because I was like*, *“well*, *damn*, *it does seem like*, *you know*, *the African American community is highest at risk*.*” And every person I’ve met*, *or*, *you know*, *come in contact with or gotten to know*, *they’ve all been positive*, *or they just contracted it*. *It’s something that I don’t necessarily wish upon anybody*, *and it sucks when*, *you know*, *somebody else has to go through the new journey for themselves*. *It’s kind of just becoming cliché now*. (Participant 12, a 29-year-old male with horizontally acquired HIV)

This shame related to confirming stereotypes was common in our sample. In fact, many participants explained that they thought that only gay men had HIV, but after attending clinic and seeing the diversity of individuals living with HIV, they were able to change their own assumptions and reduce internalized stigma. For example, one participant asserted that now, as a person living with HIV, he is able to humanize other people living with HIV:

*…Before it was like oh "person XYZ has HIV" or "person XYZ died of AIDS" and like*, *you never could relate them to a person*. *But to actually have [HIV]*, *then it’s like “oh you’re just a regular person*.*” You wake up*. *You go to sleep*. *You just take a pill every day like a daily vitamin…It’s not so like*, *"I don’t want to touch them;" "I can’t eat behind them;" "I can’t go to their house and eat with the fork that they have*.*" It’s more humanistic to me to see [others living with HIV] and to actually live with it*. (Participant 3, a 28-year-old male with horizontally acquired HIV)

Reaching acceptance of one’s own HIV status (i.e., decreasing internalized self-stigma) was described as facilitating several participants’ medication adherence and clinic attendance. One participant, for example, explained how individuals should let go of any shame they may feel about living with HIV or how it was transmitted in order to accept their diagnosis and improve their medication adherence:

*Do not be ashamed of [HIV]*. *Do not be ashamed of however you got it*. *If you got it by drugs or unprotected sex*, *don’t be ashamed of it*. *However*, *you got it*, *you now have it*. *However you got it*, *accept it*. *Don’t cry if you have it; It’s not going to go away*. *It’s not going to go away by crying […] It’s important to accept you have it and just take your medication*, *so [you don’t get sick]*. (Participant 19, a 28-year-old female with vertically acquired HIV)

### Interpersonal level

Enacted stigma was the most commonly described mechanism at the interpersonal level, including among familial relationships, peers, coworkers, and intimate partners. Several participants described how individuals living with HIV were labeled as “dirty” and “promiscuous.” When asked about how her family viewed HIV, a participant whose family were recent immigrants from Africa explained:

*It’s just you look different*. *You’re shunned*. *Just being an African person*, *the way you look at it is just*, *“How’d you get it*? *You’re a dirty person*. *You must have been a loose girl*.*” A loose girl is like you’re promiscuous*. *So that’s [why] my mom was very secretive when she found out that I had it*. *She was very secretive until her death*. (Participant 19, a 28-year-old female with vertically acquired HIV)

Similarly, stigma was at times enacted as rejection from potential and current intimate partners due to HIV status. Knowing their friends and family’s negative views of HIV, led to a fear of disclosure for some participants, including to family, friends, and intimate partners. One participant explains that given his experiences with homonegativity from family members, he has had difficulty disclosing his sexual orientation and his HIV status:

*I’ve had a family member who had it*. *It was just strictly AIDS back then and he passed away from it*, *so I honestly felt as though my family was looking very down on gay people and like “it’s nasty and you shouldn’t do that*.*” They didn’t like gay people*. *During my teen years*, *it was very hard to just kind of come out and be comfortable with just being gay*. *But also*, *just having [HIV] on top of it*, *it took a toll*, *so I decided to only just let a select few people know*. (Participant 15, a 28-year-old male with horizontally acquired HIV)

Again, stigma related to sexual orientation was often linked to HIV stigma, as illustrated by the following quote:

*A bad experience is like when I first came out as being gay*. *My family labeled me as somebody that would die from HIV*. (Participant 3, a 28-year-old male with horizontally acquired HIV)

Similar experiences were described frequently by multiple participants. In contrast to stigma operating at other ecological levels, participants were less likely to explicitly describe direct links between these interpersonal stigmatizing experiences and HIV care engagement.

### Clinic level

Stigma within healthcare settings was discussed at length by our participants, including both enacted and anticipated stigma.

#### Enacted stigma

Participants described multiple instances of enacted stigma within healthcare settings. Within the HIV clinic, a few participants recalled instances where they felt they were treated unfairly or rudely by clinic staff, which they attributed to their HIV status and sexual orientation. Participant 1 described an experience with a clinic phlebotomist:

*There are some people out there who view [HIV] as just like having leprosy*. *I’ve seen it*. *It’s just how they feel…The phlebotomist*, *[the one] who draws the blood*, *[their] attitude is horrible*. *You know*, *the last time I was there*, *we met on the stairs*, *and [they] bumped me*, *and I turned around and [they] looked at me crazy… I can’t read minds so I’m not really sure if it was necessarily because of my HIV status*, *but it felt like that*. (Participant 1, a 28-year-old male with horizontally acquired HIV)

Similarly, another participant described his experiences with a security guard within the HIV clinic:

I*t just felt like [they] didn’t want to touch what you touched*, *like*, *you know how [they] handed stuff back to you*…*[they would] throw it back*. *Like*, *you know*, *it just was very like that stigma feeling*. (Participant 13, a 29-year-old male with horizontally acquired HIV)

Several participants discussed poor treatment in healthcare settings outside of their HIV clinic, including dental offices and Emergency Departments. A few participants spoke about experiences in which their file was tagged to indicate they were a high-risk patient. For example, participant 3 explained:

*It’s like the manila folders in the dentist*. *They have manila folders and color tabs*. *It’s a red-colored tab and it’s worded higher risk or something on the tabs*. *And then you see everybody else’s folder and it’s like*, *oh*, *these are small little tabs and they’re not as big and bold and bright*. *It’s like “Wow*.” (Participant 3, a 28-year-old male with horizontally acquired HIV)

Similarly, another participant described a time when an Emergency Room doctor asked him if he could contract HIV if he drained the patients abscess:

*The doctor who was doing the incisions*, *he had read my profile*, *you know*, *of course*, *you know*, *it’s gonna pop up with my status and*, *as soon as he read that he kind of made a face*, *like he made it seem like he was saying "Ew*.*” And because me being young*, *I think I was like*, *maybe 21–22*, *so my skin was not that thick just yet for me to be able to*, *you know*, *handle somebody who’s supposed to be helping me and keeping me healthy and alive to say something like that*. *I just felt like that was just so out of line*. *And*, *you know*, *even though he didn’t say much*, *his body language spoke a lot*, *like he kept trying to wash his hands*. *And he ended up asking me [if he could get HIV from me]*, *which was kind of weird*, *because I’m like*, *you’re the doctor*, *you should know whether or not if you’ll be able to catch it if you did cut me and there was blood*, *like*, *I didn’t understand why he asked me that question*. *I was like*, *“Well*, *you should know you’re the doctor*. *You got the degree*, *not me*.*” But it was very weird*. (Participant 12, a 29-year-old male with horizontally acquired HIV)

In addition to HIV-related stigma, a few sexual minority male participants described stigma enacted by healthcare workers shaming them for sexual behaviors and practices. These encounters with healthcare workers contributed to anticipated stigma and hesitation about seeking sexual health care in the future.

*I remember I was in college*, *and I had contracted an STD…I went to the [name of clinic]*, *and I just really felt judged*, *because the lady was just like*, *basically saying*, *I should know better out here having sex unprotective and stuff like that*. *And I just felt like from a healthcare worker*, *if I’m coming to you not knowing anything*, *or discovering something about me*, *or even if I do know*, *it’s already embarrassing enough so for a healthcare worker to tell you like*, *"oh*, *you should know better*…*like ew what’s this*,*” it makes you uncomfortable*. *It makes you not ever want to go to somebody*. (Participant 8, a 28-year-old male with horizontally acquired HIV)

Those who brought up racial stigma expressed uncertainty around whether their negative treatment in healthcare settings was due to race. Racial stigma in healthcare settings was enacted through experiences of being overlooked; for example having to wait longer than White individuals for treatment, or having healthcare workers not believe their pain status, especially in emergency rooms.

*When I was at the [private hospital] emergency room*, *I was waiting forever*. *I felt really bad*. *I was waiting to be seen*. *I was waiting to go into the room while everybody else that was white got into a room… I have to wait for a long period to be seen*, *unless I’ve gotten hit by a car*. (Participant 19, a 29-year-old female with vertically acquired HIV)

#### Anticipated stigma

Participants explained that these experiences with enacted stigma related to sexual behaviors also contributed directly to decreased engagement in HIV care due in part to the development of anticipated stigma. Participant 8 (a 28-year-old male with horizontally acquired HIV) explained that after feeling judged by a healthcare provider for contracting an STD, he stopped taking his medications and coming to clinic:

*It impacted me a little bit because I had stopped taking [my HIV medications] for a little moment because I was feeling a little sad after hearing the [doctor judge me for having an STI]*…*even though it had nothing to do with my HIV*, *it was just like*, *made me feel sad that someone could judge me off something*. *I would [previously] go frequently to my appointments [at the HIV clinic]*, *but after that*, *I did kind of drop off from going because like I said*, *I ain’t want to hear that from another healthcare worker so I was just like*, *“Let me just kind of lay back a little*.”

Anticipated stigma was also described as a barrier to disclosing HIV status in non-HIV healthcare settings. Disclosing HIV status to providers outside of the HIV clinic was stressful for some participants such as participant 5 (a 27-year-old female with horizontally acquired HIV), who avoided sharing her status with dental providers because she did not know what they would think or say behind her back. Others explained that they similarly did not disclose their status in non-HIV healthcare settings because they did not want to be treated poorly.

*In the beginning—the first year–yes*, *I did avoid telling the dentist [my HIV status] and I tried not to go to the hospital*. *[I was afraid] they were gonna treat me differently*. *I just thought they would be treating me differently and that I would be in a different area of the doctor’s office […] I didn’t know what they would be thinking in their heads or what they would say when they went back to the office*. (Participant 5, a 27-year-old female with horizontally acquired HIV)

In contrast, a few participants discussed how they felt less anxious and less stigmatized when attending the HIV clinic compared to other clinics because of the diversity of patients within the waiting rooms. The experience with their clinic peers helped decrease anxiety for several participants, as participant 5 explains (a 27-year-old female with horizontally acquired HIV):

*Everyone would be different*. *I see different races*, *different types of people—men*…*women—all of that*…*so I just felt more comfortable because I saw different people there*. *And they weren’t like hiding themselves*, *they were just sitting there calmly*.

### Community level

Experiences with enacted stigma were extensively described at the community level throughout the participant interviews, who specifically described harmful community norms and views relating to HIV and sexuality expressed by various groups and institutions including employers, community leaders, religious community, and within the media. Several participants expressed their view that the Black community in particular views same-sex behavior as being synonymous with HIV.

*I felt stereotyped*, *if I should say*, *because*, *you know*, *in a Black community*, *like in the straight Black community*, *you know*, *especially with older people in church*, *that’s what they think*. *They think*, *oh*, *you’re gay*, *you’re gonna have AIDS*. (Participant 2, a 28-year-old male with horizontally acquired HIV)

Similarly, many participants described how people in their communities associate HIV with stigmatized sexual behaviors and promiscuity. This finding echoed discussions of stigma experienced at the interpersonal level. When asked how HIV is viewed in his community, participant 1 (a 28-year-old male with horizontally acquired HIV) explained:

*Kind of like leprosy*. *Kind of like something nasty*. *Assumptions are made about your sexual escapades and how you conduct yourself*. *A lot of that is based on your HIV status*. *I hear the term ‘clean’ used a lot and that used to sting because I’ve never been someone who was promiscuous*.

### Coping and motivating mechanisms

Participants described motivating factors and strategies for coping with different types of stigma related to their HIV status, race, sexual orientation, and/or sexual behaviors, including prioritizing one’s health, social support, and affirming experiences with the medical care team.

#### Prioritizing health

Some participants felt that staying engaged in care was the best coping mechanism for HIV stigma.

*For someone like myself*, *[HIV stigma] kind of made me feel motivated to like*, *make sure I stayed on top of my health*, *and to do my best to take control of this virus*. (Participant 11, a 28-year-old male with horizontally acquired HIV)

Some participants further discussed how such care engagement and health maintenance would in turn raise awareness for others that people living with HIV can also be healthy. Interestingly, a few participants reported that they were motivated to become more engaged in their HIV care, including taking their medications and attending clinic, to overcome non-HIV related stigma experienced in the community.

*I really just realized that care and treatment is the best thing I can do to combat stigma [about my race and sexual orientation]…to take care of myself which ultimately will teach the people around me and then it will raise awareness for people who don’t know much about [HIV] to learn that there are more people who are taking care of themselves who are healthy and who may have contracted HIV*. (Participant 18, a 28-year-old male with horizontally acquired HIV)

Similarly, another participant explained that experiences with stigma related to his race and sexual orientation impacted his HIV care by motivating him to be more engaged in his HIV care:

*So I stayed in [name of Midwestern state] for a month for work*, *and I experienced stigma for things outside of being HIV positive*, *because it was the first time literally someone looked at my skin color and I felt it*. *It was the first time that someone looked at my sexuality and I felt it*. *[…] It impacted my HIV care because it made me want to be more vigilant about myself*. *Whatever I had to do to keep myself at 100% or 1*,*000% or more in order to keep fighting [racism and homophobia] outside of HIV*, *that’s what I’m going to do*. *[It] pushes me to make sure that internally I’m always going to be okay*. (Participant 6, a 29-year-old male with horizontally acquired HIV)

#### Social support

Other coping mechanisms described by the participants include religion, peer/family support, self-motivation, and good patient-provider communication. Additionally, utilization of mental health services provided in their HIV clinic helped participants overcome internalized stigma, including improving participant’s self-esteem.

*Therapy helped with just like higher self-esteem and accepting my flaws and HIV more and embracing them instead of hating them*. *Like this is who I am*, *I don’t have to pretend or put on*…*this who you are*. *If you don’t like something you can change it*, *but be accepting and love yourself as you are first*. (Participant 1, a 28-year-old male with horizontally acquired HIV)

Furthermore, coping with negative feelings about living with HIV seems to change over time. Patients describe that as they get older, they feel taking their medication is important not only for themselves, but for their families, friends, sexual partners, and/or future selves. One participant explained that although they sometimes get tired of taking their medications, they will continue to due to recurring thoughts about staying healthy in the future.

*There are moments where I don’t want to [take my HIV medicines] at all*. *I feel as though ‘this is stupid*, *this is dumb*.*’ I have thoughts like that sometimes*, *like why do we have to go through this*? *Why can’t we just be normal people and just feel normal*, *and why did diseases have to come into play*? *And then it’s just heavy on my heart… If I didn’t take it*, *I could be in a horrible situation where I could be sick in a hospital*, *or I could be dead*, *and I don’t want that for myself*. *I wouldn’t want that for anybody else*, *so that really gives me the motivation to take [my medicines] every day*, *take it when I’m supposed to take it on time*. (Participant 15, a 28-year-old male with horizontally acquired HIV)

#### Role of the medical care team

Several participants detailed affirming experiences with medical care teams in pediatric clinic, including doctors and social workers, which helped them cope with HIV stigma after their initial diagnosis while also addressing and being sensitive to traumas related to sexual orientation stigma.

*In the beginning [after I was first diagnosed]*, *it really was my pediatric doctors who helped me deal [with stigma]*. *And I had a social worker who was really dedicated to making sure that I was okay*, *because [they] knew about the verbal abuse [surrounding my sexual orientation] when I was younger*. *But [they were] really attentive to a lot of things that went on in my household*, *and*, *you know*, *for me*, *it was kind of refreshing to have just certain male and female figures*, *you know*, *just actually showing that they were concerned*. (Participant 12, a 29-year-old male with horizontally acquired HIV)

Moreover, open, honest communication with medical providers, including about enacted stigma experienced within the clinic, helped participants stay engaged in care.

*[My experience with stigma from a clinic staff member] did not affect me because I talked to my doctor…It would have affected me because I don’t want somebody that is drawing blood from me to hurt me or*, *you know*, *to not have that care for me*. *Talking to my doctor helped me understand that I just need to do what I have to do to stay healthy*. (Participant 1, 28-year-old male with horizontally acquired HIV)

In contrast, some participants discussed that in order to cope with anticipated stigma within the HIV clinic, they feel like they need to “put on another face” and “shut their emotions off and operate on auto-pilot.” These participants described how they become emotionally detached and sometimes avoid coming to the clinic altogether to protect themselves from potential shame or discrimination when coming to clinic.

## Discussion

Participants’ experiences of stigma within various social environments significantly shaped their lived experience and their engagement in HIV care. While these stigmas have previously been documented in other populations living with HIV [14, 16, 28–30], our study adds to these findings by focusing on stigma mechanisms relevant to varying social contexts of young adults living with HIV, and by shedding light on coping and motivating strategies. While prior work has focused more on individual and community levels, our study primarily highlighted instances of stigma being enacted and anticipated in healthcare settings. We found that the salience of different stigma mechanisms and coping/motivating factors varied by socio-ecological level; these patterns have potential implications for intervention.

At the intrapersonal level, prior studies have shown that intrapersonal HIV stigma is associated with lower levels of viral suppression, due in part to feelings of shame and anticipation of being stigmatized when accessing services [31–34]. Participants in our study similarly described feelings of shame surrounding their HIV status that was at times compounded by shame surrounding confirming stereotypes about Black gay men and HIV. These intersecting intrapersonal stigmas were associated with delayed care seeking, isolation, and fear of disclosure. Self-isolation associated with internalized HIV stigma was similarly seen in a qualitative study among persons living with HIV in China [35]. In Xie et al., participants internalized familial and cultural beliefs about HIV which led to self-blame and self-isolation due to low self-worth, as a way to cope with their existing internalized stigma, but also as a way to “escape anticipated stigma and discrimination” [35]. Reassuring HIV knowledge and exposure to a variety of people with different races, genders, and sexual orientation living with HIV after they were diagnosed eased participants’ concerns about HIV stereotypes and self-blame. Additionally, the knowledge of life experiences of other people living with HIV helped some participants cope with internalized stigma and decreased their need for isolation. Future interventions should incorporate peer support and HIV knowledge to mitigate intrapersonal intersecting stigmas.

At the interpersonal level, discrimination from family and friends about HIV, race, and/or sexuality contributed to avoidance of close relationships, in part to avoid serostatus disclosure. Negative feelings and misconceptions about HIV, race, and sexual orientation were common from participants family members even prior to diagnosis. Interestingly, we also found that some participants avoided intimate relationships because they remembered their own stigma towards HIV prior to their seroconversion. This suggests that different forms of stigma (e.g., internalized, enacted, and anticipated stigma) may be contributing to a fear of disclosure and self-isolation. Family and partner-based interventions addressing unity, trust, and HIV knowledge may help with HIV status disclosure and decrease interpersonal stigmas.

At the clinic level, our findings are supported by previous studies demonstrating that medical mistrust and discrimination from providers contributes to suboptimal HIV care outcomes. For example, Cooper et al. found that Black patients reported receiving poorer care from and having less confidence in providers with higher implicit prejudice [36]. In our study, participants similarly described experiencing different forms of intersecting stigmas in healthcare settings, including within HIV clinics. These experiences were described as barriers to clinic attendance and medication adherence. These findings support the results of previous studies that found that institutional and structural marginalization of young men who have sex with men and transgender women compounds HIV stigma as a barrier to care [37, 38]. This idea that clinic-level factors such as staff attitudes regarding homophobia and racism and lack of culturally competent providers may perpetuate feelings of stigma in young adults living with HIV expanding upon prior work by Philbin et al. [39] and others [37, 40]. Additionally, our study shows that co-existing stigmas impact patient-provider interactions and feelings of medical mistrust, contributing to young adults being less willing to seek out various forms of medical services, including STI testing and treatment, dental care, and routine HIV care. This suggests that providers and staff in both HIV and other healthcare settings will need to be aware of how different types of stigma contributes to feelings of marginalization and decreased care utilization. In addition, previous studies have shown that structural competency training for healthcare professionals, including primary care physicians caring for individuals with opioid use disorder, can help provide guidance on how to recognize and address structural factors that lead to different types of stigma and health inequities [41–43]. Thus, a structural competency curriculum for providers and healthcare staff could be used to provide more understanding of the structures that are shaping the experiences of young adults living with HIV and their communities and ultimately provide a more integrated and patient-centered approach to care. Future interventions, including qualitative improvement efforts, can address structural competency and decrease stigma among providers and staff within healthcare settings.

At the community level, we found that participants experienced discrimination related to HIV, race, and sexuality by various groups and institutions including employers, community leaders, the religious community, and within the media. Our findings are supported by previous studies that suggest that Black men and women experience intersecting stigmas related to HIV, race, gender, and socioeconomic class in certain geographic locations, such as in the Southern United States, at the community and institutional level [44–46]. Interestingly, while we found that most participants described instances of enacted stigma in their communities, prior research by Turan et al. found that perceived community stigma by adults living with HIV was mediated by internalized and anticipated stigma and associated with decreased medication adherence [47]. Although previous research has described spirituality and religion as a coping strategy for internalized HIV stigma experienced by older women living with HIV in the South, our study indicated that young adults living with HIV experienced enacted stigma in the religious community. Future community-based interventions such as peer-based media campaigns targeting intersecting stigmas, HIV knowledge, and social support for young adults living with HIV have the potential to mitigate and/or prevent experiences with enacted stigma.

Finally, participants’ descriptions of coping and motivating strategies give further insight into potential anti-stigma strategies. Most importantly, social support from HIV clinic providers, peers, and family helped some young adults cope with different forms of stigma and related adverse mental health consequences. This aligns with recent research that suggests that social support may buffer the negative effects of HIV-related stigma on clinical outcomes in both women with HIV and Black adults living with HIV [48, 49]. Additionally, some participants in this study described the resilience that developed at the intersection of multiple identities, including the desire and motivation to improve their engagement in HIV care to overcome deep-rooted societal/community racial stigma. To date, the possible relationships between social support and intersectional stigma has not been specifically studied, especially in young adults living with HIV. Our research demonstrates a need to further elucidate and develop social support interventions for young adults living with HIV with specific attention to intersecting identities and stigmas.

### Limitations

While we intentionally made efforts to include young adults with vertically acquired HIV, as well as women, the majority of participants in the parent study (and thus in our substudy as well) were young Black men with horizontally acquired HIV. Larger numbers of participants from other groups (e.g., cisgender women, adolescents and young adults with perinatally acquired infection) might have yielded additional types of experiences or narratives relating to intersectional stigma. However, our study population distribution is reflective of US epidemiology, as young Black gay, bisexual and other men who have sex with men bear a disproportionate burden of HIV relative to other groups [50]. It is also important to note that individuals who agreed to participate in our study may have different experiences with stigma from those who did not. That is, those who had higher internalized stigma or less effective strategies may have been less likely to want to participate in HIV-related research. Additionally, researchers’ personal beliefs or preconceived notions may unconsciously have shaped their observations or conclusions when interpreting participant’s responses. In order to minimize this limitation, we intentionally had multiple study team members from different backgrounds independently code transcripts, establish inter-coder reliability and discuss interpretation of findings.

## Conclusions

Our participants described experiences with intersecting stigmas, and delineated connections between these experiences and their own HIV care engagement. This study addressed several critical gaps in HIV-related stigma research and suggests areas for improvement. Future interventions aiming to improve care engagement for young adults living with HIV should address the different mechanisms of stigma at the community, clinic, interpersonal and intrapersonal levels by enhancing social support and improving healthcare structural competency. Future research to develop and implement such interventions is needed to improve the health and well-being for young adults living with HIV, in order to address health inequities and move towards ending the HIV epidemic in the US.

## Supporting information

S1 Appendix Consolidated criteria for reporting qualitative research (COREQ) checklist. (DOCX)

## References

[1] Centers for Diseases Control and Prevention. Estimated HIV incidence and prevalence in the United States, 2014–2018. HIV Surveillance Supplemental Report. 2020;25.

[2] Philbin MM, Tanner AE, Chambers BD, Ma A, Ware S, Lee S, et al. Transitioning HIV-infected adolescents to adult care at 14 clinics across the United States: using adolescent and adult providers’ insights to create multi-level solutions to address transition barriers. AIDS Care. 2017;29(10):1227–34. doi: 10.1080/09540121.2017.1338655 28599596 PMC5573205

[3] Zanoni BC, Mayer KH. The adolescent and young adult HIV cascade of care in the United States: exaggerated health disparities. AIDS Patient Care STDS. 2014;28(3):128–35. doi: 10.1089/apc.2013.0345 24601734 PMC3948479

[4] Katz IT, Ryu AE, Onuegbu AG, Psaros C, Weiser SD, Bangsberg DR, et al. Impact of HIV-related stigma on treatment adherence: systematic review and meta-synthesis. J Int AIDS Soc. 2013;16(3 Suppl 2):18640. doi: 10.7448/IAS.16.3.18640 24242258 PMC3833107

[5] Martinez J, Harper G, Carleton RA, Hosek S, Bojan K, Clum G, et al. The impact of stigma on medication adherence among HIV-positive adolescent and young adult females and the moderating effects of coping and satisfaction with health care. AIDS Patient Care STDS. 2012;26(2):108–15. doi: 10.1089/apc.2011.0178 22149767 PMC3266519

[6] Enane LA, Apondi E, Toromo J, Bosma C, Ngeresa A, Nyandiko W, et al. "A problem shared is half solved"—a qualitative assessment of barriers and facilitators to adolescent retention in HIV care in western Kenya. AIDS Care. 2020;32(1):104–12. doi: 10.1080/09540121.2019.1668530 31554414 PMC6883166

[7] Toromo JJ, Apondi E, Nyandiko WM, Omollo M, Bakari S, Aluoch J, et al. "I have never talked to anyone to free my mind"—challenges surrounding status disclosure to adolescents contribute to their disengagement from HIV care: a qualitative study in western Kenya. BMC Public Health. 2022;22(1):1122. doi: 10.1186/s12889-022-13519-9 35658924 PMC9167528

[8] Goffman E. Stigma: Notes on the Management of Spoiled Identity. New York, New York: Simon & Schuster, Inc.; 1963.

[9] Earnshaw VA, Bogart LM, Dovidio JF, Williams DR. Stigma and racial/ethnic HIV disparities: moving toward resilience. Am Psychol. 2013;68(4):225–36. doi: 10.1037/a0032705 23688090 PMC3740715

[10] Earnshaw VA, Chaudoir SR. From conceptualizing to measuring HIV stigma: a review of HIV stigma mechanism measures. AIDS Behav. 2009;13(6):1160–77. doi: 10.1007/s10461-009-9593-3 19636699 PMC4511707

[11] Rueda S, Mitra S, Chen S, Gogolishvili D, Globerman J, Chambers L, et al. Examining the associations between HIV-related stigma and health outcomes in people living with HIV/AIDS: a series of meta-analyses. BMJ Open. 2016;6(7):e011453. doi: 10.1136/bmjopen-2016-011453 27412106 PMC4947735

[12] Crenshaw K. Demarginalizing the Intersection of Race and Sex: A Black Feminist Critique of Antidiscrimination Doctrine, Feminist Theory and Antiracist Politics. University of Chicago Legal Forum [Internet]. 1989; 1989(1). Available from: https://chicagounbound.uchicago.edu/uclf/vol1989/iss1/8.

[13] Algarin AB, Zhou Z, Cook CL, Cook RL, Ibanez GE. Age, Sex, Race, Ethnicity, Sexual Orientation: Intersectionality of Marginalized-Group Identities and Enacted HIV-Related Stigma Among People Living with HIV in Florida. AIDS Behav. 2019;23(11):2992–3001. doi: 10.1007/s10461-019-02629-y 31392442 PMC6803104

[14] Logie CH, James L, Tharao W, Loutfy MR. HIV, gender, race, sexual orientation, and sex work: a qualitative study of intersectional stigma experienced by HIV-positive women in Ontario, Canada. PLoS Med. 2011;8(11):e1001124. doi: 10.1371/journal.pmed.1001124 22131907 PMC3222645

[15] Rai SS, Peters RMH, Syurina EV, Irwanto I, Naniche D, Zweekhorst MBM. Intersectionality and health-related stigma: insights from experiences of people living with stigmatized health conditions in Indonesia. Int J Equity Health. 2020;19(1):206. doi: 10.1186/s12939-020-01318-w 33176809 PMC7661268

[16] Rice WS, Logie CH, Napoles TM, Walcott M, Batchelder AW, Kempf MC, et al. Perceptions of intersectional stigma among diverse women living with HIV in the United States. Soc Sci Med. 2018;208:9–17. doi: 10.1016/j.socscimed.2018.05.001 29753137 PMC6015551

[17] Bronfenbrenner U. Toward an experimental ecology of human development. American psychologist. 1977;32(7):513.

[18] Stokols D. Translating social ecological theory into guidelines for community health promotion. Am J Health Promot. 1996;10(4):282–98. doi: 10.4278/0890-1171-10.4.282 10159709

[19] Sandelowski M, Lambe C, Barroso J. Stigma in HIV-positive women. J Nurs Scholarsh. 2004;36(2):122–8. doi: 10.1111/j.1547-5069.2004.04024.x 15227758

[20] Embleton L, Logie CH, Ngure K, Nelson L, Kimbo L, Ayuku D, et al. Intersectional Stigma and Implementation of HIV Prevention and Treatment Services for Adolescents Living with and at Risk for HIV: Opportunities for Improvement in the HIV Continuum in Sub-Saharan Africa. AIDS Behav. 2023;27(Suppl 1):162–84. doi: 10.1007/s10461-022-03793-4 35907143 PMC10192191

[21] Halyard AS, Doraivelu K, Camacho-Gonzalez AF, Del Rio C, Hussen SA. Examining healthcare transition experiences among youth living with HIV in Atlanta, Georgia, USA: a longitudinal qualitative study. J Int AIDS Soc. 2021;24(2):e25676. doi: 10.1002/jia2.25676 33619890 PMC7900438

[22] Hussen SA, Chakraborty R, Knezevic A, Camacho-Gonzalez A, Huang E, Stephenson R, et al. Transitioning young adults from paediatric to adult care and the HIV care continuum in Atlanta, Georgia, USA: a retrospective cohort study. J Int AIDS Soc. 2017;20(1):21848. doi: 10.7448/IAS.20.1.21848 28872281 PMC5705166

[23] Hussen SA, Doraivelu K, Goldstein MH, Shenvi N, Easley KA, Zanoni BC, et al. Human Immunodeficiency Virus (HIV) Care Continuum Outcomes After Transition to Adult Care Among a Prospective Cohort of Youth With HIV in Atlanta, Georgia. Clin Infect Dis. 2023;76(7):1218–24. 36409586 10.1093/cid/ciac904 PMC10319754

[24] Tong A, Sainsbury P, Craig J. Consolidated criteria for reporting qualitative research (COREQ): a 32-item checklist for interviews and focus groups. Int J Qual Health Care. 2007;19(6):349–57. doi: 10.1093/intqhc/mzm042 17872937

[25] Braun V, Clarke V. Using thematic analysis in psychology. Qualitative Research in Psychology. 2006;3(77):77–101.

[26] Burla L, Knierim B, Barth J, Liewald K, Duetz M, Abel T. From text to codings: intercoder reliability assessment in qualitative content analysis. Nurs Res. 2008;57(2):113–7. doi: 10.1097/01.NNR.0000313482.33917.7d 18347483

[27] Hennink M, Hutter I, Bailey A. Qualitative Research Methods: Sage; 2010.

[28] Yannessa JF, Reece M, Basta TB. HIV provider perspectives: the impact of stigma on substance abusers living with HIV in a rural area of the United States. AIDS Patient Care STDS. 2008;22(8):669–75. doi: 10.1089/apc.2007.0151 18627281

[29] Brinkley-Rubinstein L. Understanding the Effects of Multiple Stigmas Among Formerly Incarcerated HIV-Positive African American Men. AIDS Educ Prev. 2015;27(2):167–79. doi: 10.1521/aeap.2015.27.2.167 25915701

[30] Ogunbajo A, Iwuagwu S, Williams R, Biello KB, Kahler CW, Sandfort TGM, et al. Experiences of minority stress among gay, bisexual, and other men who have sex with men (GBMSM) in Nigeria, Africa: The intersection of mental health, substance use, and HIV sexual risk behavior. Glob Public Health. 2021;16(11):1696–710. doi: 10.1080/17441692.2020.1834598 33108249 PMC8076332

[31] Hargreaves JR, Pliakas T, Hoddinott G, Mainga T, Mubekapi-Musadaidzwa C, Donnell D, et al. HIV Stigma and Viral Suppression Among People Living With HIV in the Context of Universal Test and Treat: Analysis of Data From the HPTN 071 (PopART) Trial in Zambia and South Africa. J Acquir Immune Defic Syndr. 2020;85(5):561–70. doi: 10.1097/QAI.0000000000002504 32991336 PMC7654947

[32] Lorenc T, Marrero-Guillamon I, Llewellyn A, Aggleton P, Cooper C, Lehmann A, et al. HIV testing among men who have sex with men (MSM): systematic review of qualitative evidence. Health Educ Res. 2011;26(5):834–46. doi: 10.1093/her/cyr064 21873612

[33] Quinn K, Voisin DR, Bouris A, Jaffe K, Kuhns L, Eavou R, et al. Multiple Dimensions of Stigma and Health Related Factors Among Young Black Men Who Have Sex with Men. AIDS Behav. 2017;21(1):207–16. doi: 10.1007/s10461-016-1439-1 27233249 PMC5124546

[34] Kemp CG, Lipira L, Huh D, Nevin PE, Turan JM, Simoni JM, et al. HIV stigma and viral load among African-American women receiving treatment for HIV. AIDS. 2019;33(9):1511–9. doi: 10.1097/QAD.0000000000002212 31259767 PMC6621603

[35] Xie T, Yang JP, Simoni JM, Shiu CS, Chen WT, Zhao H, et al. Unable to be a Human Being in Front of Other People: A Qualitative Study of Self-Isolation Among People Living with HIV/AIDS in China. J Clin Psychol Med Settings. 2017;24(3–4):211–22. doi: 10.1007/s10880-017-9513-z 29086186 PMC5709208

[36] Cooper LA, Roter DL, Carson KA, Beach MC, Sabin JA, Greenwald AG, et al. The associations of clinicians’ implicit attitudes about race with medical visit communication and patient ratings of interpersonal care. Am J Public Health. 2012;102(5):979–87. doi: 10.2105/AJPH.2011.300558 22420787 PMC3483913

[37] Arrington-Sanders R, Hailey-Fair K, Wirtz AL, Morgan A, Brooks D, Castillo M, et al. Role of Structural Marginalization, HIV Stigma, and Mistrust on HIV Prevention and Treatment Among Young Black Latinx Men Who Have Sex with Men and Transgender Women: Perspectives from Youth Service Providers. AIDS Patient Care STDS. 2020;34(1):7–15. doi: 10.1089/apc.2019.0165 31944853 PMC6983743

[38] Beach LB, Greene GJ, Lindeman P, Johnson AK, Adames CN, Thomann M, et al. Barriers and Facilitators to Seeking HIV Services in Chicago Among Young Men Who Have Sex with Men: Perspectives of HIV Service Providers. AIDS Patient Care STDS. 2018;32(11):468–76. doi: 10.1089/apc.2018.0094 30398956 PMC6247379

[39] Philbin MM, Tanner AE, DuVal A, Ellen JM, Kapogiannis B, Fortenberry JD, et al. Understanding Care Linkage and Engagement Across 15 Adolescent Clinics: Provider Perspectives and Implications for Newly HIV-Infected Youth. AIDS Educ Prev. 2017;29(2):93–104. doi: 10.1521/aeap.2017.29.2.93 28467164 PMC5441680

[40] Lichtenstein B. Stigma as a barrier to treatment of sexually transmitted infection in the American deep south: issues of race, gender and poverty. Soc Sci Med. 2003;57(12):2435–45. doi: 10.1016/j.socscimed.2003.08.002 14572849

[41] Metzl JM, Hansen H. Structural competency: theorizing a new medical engagement with stigma and inequality. Soc Sci Med. 2014;103:126–33. doi: 10.1016/j.socscimed.2013.06.032 24507917 PMC4269606

[42] Bagchi AD. A Structural Competency Curriculum for Primary Care Providers to Address the Opioid Use Disorder, HIV, and Hepatitis C Syndemic. Front Public Health. 2020;8:210. doi: 10.3389/fpubh.2020.00210 32582612 PMC7289946

[43] Caiola C, Nelson TB, Black KZ, Calogero C, Guard K, Haberstroh A, et al. Structural competency in pre-health and health professional learning: A scoping review. J Interprof Care. 2022:1–10. doi: 10.1080/13561820.2022.2124238 36264080 PMC10188213

[44] Blake BJ, Taylor GA, Sowell RL. Exploring Experiences and Perceptions of Older African American Males Aging With HIV in the Rural Southern United States. Am J Mens Health. 2017;11(2):221–32. doi: 10.1177/1557988316662875 27550774 PMC5675302

[45] Caiola C, Barroso J, Docherty SL. Capturing the Social Location of African American Mothers Living With HIV: An Inquiry Into How Social Determinants of Health Are Framed. Nurs Res. 2017;66(3):209–21. doi: 10.1097/NNR.0000000000000213 28252555 PMC5407908

[46] Fletcher F, Ingram LA, Kerr J, Buchberg M, Bogdan-Lovis L, Philpott-Jones S. "She Told Them, Oh That Bitch Got AIDS": Experiences of Multilevel HIV/AIDS-Related Stigma Among African American Women Living with HIV/AIDS in the South. AIDS Patient Care STDS. 2016;30(7):349–56. doi: 10.1089/apc.2016.0026 27410498 PMC4948216

[47] Turan B, Budhwani H, Fazeli PL, Browning WR, Raper JL, Mugavero MJ, et al. How Does Stigma Affect People Living with HIV? The Mediating Roles of Internalized and Anticipated HIV Stigma in the Effects of Perceived Community Stigma on Health and Psychosocial Outcomes. AIDS Behav. 2017;21(1):283–91. doi: 10.1007/s10461-016-1451-5 27272742 PMC5143223

[48] Cuca YP, Asher A, Okonsky J, Kaihura A, Dawson-Rose C, Webel A. HIV Stigma and Social Capital in Women Living With HIV. J Assoc Nurses AIDS Care. 2017;28(1):45–54. doi: 10.1016/j.jana.2016.09.001 27697368 PMC5183462

[49] Galvan FH, Davis EM, Banks D, Bing EG. HIV stigma and social support among African Americans. AIDS Patient Care STDS. 2008;22(5):423–36. doi: 10.1089/apc.2007.0169 18373417 PMC2831751

[50] Wejnert C, Hess KL, Rose CE, Balaji A, Smith JC, Paz-Bailey G, et al. Age-Specific Race and Ethnicity Disparities in HIV Infection and Awareness Among Men Who Have Sex With Men—20 US Cities, 2008–2014. J Infect Dis. 2016;213(5):776–83. doi: 10.1093/infdis/jiv500 26486637 PMC6281353

